# Targeting *aldose reductase* using natural African compounds as promising agents for managing diabetic complications

**DOI:** 10.3389/fbinf.2025.1499255

**Published:** 2025-02-06

**Authors:** Miriam E. L. Gakpey, Shadrack A. Aidoo, Toheeb A. Jumah, George Hanson, Siyabonga Msipa, Florence N. Mbaoji, Omonijo Bukola, Palesa C. Tjale, Mamadou Sangare, Hedia Tebourbi, Olaitan I. Awe

**Affiliations:** ^1^ Department of Clinical Pathology, Noguchi Memorial Institute for Medical Research, University of Ghana, Legon, Ghana; ^2^ Department of Virology, Noguchi Memorial Institute for Medical Research, University of Ghana, Legon, Ghana; ^3^ School of Collective Intelligence, University Mohammed VI Polytechnic, Rabat, Morocco; ^4^ Department of Parasitology, Noguchi Memorial Institute for Medical Research, University of Ghana, Legon, Ghana; ^5^ Department of Integrative Biomedical Science, Faculty of Health Sciences, University of Cape Town, Cape Town, South Africa; ^6^ Department of Pharmacology and Toxicology, Faculty of Pharmaceutical Sciences, University of Nigeria, Nsukka, Enugu, Nigeria; ^7^ Department of Medical Laboratory Science, Faculty of Basic Medical Sciences, Ladoke Akintola University of Technology, Ogbomosho, Oyo, Nigeria; ^8^ Department of Computational Biology, Faculty of Health Sciences, University of Cape Town, Cape Town, South Africa; ^9^ African Center of Excellence in Bioinformatics (ACE-B), University of Science, Techniques and Technologies of Bamako (USTTB), Bamako, Mali; ^10^ Pathophysiology, Food and Biomolecules Laboratory, Higher Institute of Biotechnology of Sidi Thabet, Sidi Thabet, Tunisia; ^11^ African Society for Bioinformatics and Computational Biology, Cape Town, South Africa

**Keywords:** diabetes mellitus, aldose reductase, molecular docking, pharmacokinetics, molecular dynamics simulations

## Abstract

**Background:**

Diabetes remains a leading cause of morbidity and mortality due to various complications induced by hyperglycemia. Inhibiting Aldose Reductase (AR), an enzyme that converts glucose to sorbitol, has been studied to prevent long-term diabetic consequences. Unfortunately, drugs targeting AR have demonstrated toxicity, adverse reactions, and a lack of specificity. This study aims to explore African indigenous compounds with high specificity as potential AR inhibitors for pharmacological intervention.

**Methodology:**

A total of 7,344 compounds from the AfroDB, EANPDB, and NANPDB databases were obtained and pre-filtered using the Lipinski rule of five to generate a compound library for virtual screening against the Aldose Reductase. The top 20 compounds with the highest binding affinity were selected. Subsequently, *in silico* analyses such as protein-ligand interaction, physicochemical and pharmacokinetic profiling (ADMET), and molecular dynamics simulation coupled with binding free energy calculations were performed to identify lead compounds with high binding affinity and low toxicity.

**Results:**

Five natural compounds, namely, (+)-pipoxide, Zinc000095485961, Naamidine A, (−)-pipoxide, and 1,6-di-o-p-hydroxybenzoyl-beta-d-glucopyranoside, were identified as potential inhibitors of aldose reductase. Molecular docking results showed that these compounds exhibited binding energies ranging from −12.3 to −10.7 kcal/mol, which were better than the standard inhibitors (zopolrestat, epalrestat, IDD594, tolrestat, and sorbinil) used in this study. The ADMET and protein-ligand interaction results revealed that these compounds interacted with key inhibiting residues through hydrogen and hydrophobic interactions and demonstrated favorable pharmacological and low toxicity profiles. Prediction of biological activity highlighted Zinc000095485961 and 1,6-di-o-p-hydroxybenzoyl-beta-d-glucopyranoside as having significant inhibitory activity against aldose reductase. Molecular dynamics simulations and MM-PBSA analysis confirmed that the compounds bound to AR exhibited high stability and less conformational change to the AR-inhibitor complex.

**Conclusion:**

This study highlighted the potential inhibitory activity of 5 compounds that belong to the African region: (+)-Pipoxide, Zinc000095485961, Naamidine A, (−)-Pipoxide, and 1,6-di-o-p-hydroxybenzoyl-beta-d-glucopyranoside. These molecules inhibiting the aldose reductase, the key enzyme of the polyol pathway, can be developed as therapeutic agents to manage diabetic complications. However, we recommend *in vitro* and *in vivo* studies to confirm our findings.

## 1 Introduction

Diabetes Mellitus (DM) is a chronic metabolic disorder characterized by hyperglycemia due to absolute lack, inadequate insulin production, or insulin resistance (i.e., the cells of the body become unresponsive to the insulin’s effects) (Sapra and Bhandari, 2021). Globally, the incidence of diabetes mellitus has surged to epidemic levels, especially in lower and middle-income countries. According to the 2021 International Diabetes Federation (IDF) report, DM affects approximately 575 million adults (20–79 years), who make up 10.5% of the world’s population, and is the seventh leading cause of death worldwide (Vos et al., 2012; IDF Diabetes Atlas, 2021). The complications associated with diabetes include micro- and macrovascular damage (such as diabetic nephropathy), retinopathy, and neuropathy. In addition to these common complications, emerging issues like cancer, liver disease, and cognitive disability also contribute to deaths associated with diabetes (Tomic et al., 2022). Previous studies have examined possible factors linked to the risk of type 1 diabetes (Abolo et al., 2024). High glucose levels in both type 1 and type 2 diabetes activate several metabolic pathways, producing toxic byproducts, which cause pathological and functional changes in various tissues (Forbes and Cooper, 2013). One of the metabolic pathways identified to contribute to the development of many diabetic consequences is the polyol pathway (Srikanth and Orrick, 2022).

The polyol pathway is a two-step metabolic pathway involved in converting glucose to sorbitol through the action of aldose reductase (AR), followed by the conversion of sorbitol to fructose via sorbitol dehydrogenase (Low, 2005). Usually, below normal glucose concentrations, most cellular glucose is directed toward the glycolytic pathway, with only a fraction entering the polyol pathway. However, in hyperglycemic conditions, such as that observed in diabetes, there is a notable increase in the flux through the polyol pathway, accounting for over thirty percent of glucose metabolism (Tang et al., 2012). This heightened activity of the polyol pathway under elevated glucose levels results in a substantial diversion of glucose towards sorbitol production, facilitated by AR, at the expense of cellular nicotinamide adenine dinucleotide phosphate (NADPH), which is a cofactor of AR (Singh et al., 2021a; Gupta, 2023). Given the essential role of NADPH in generating glutathione (GSH), an intracellular antioxidant, the depletion of NADPH by the AR can compromise the cellular antioxidant defense mechanism. Subsequently, sorbitol is converted to fructose by sorbitol dehydrogenase, accompanied by the generation of nicotinamide adenine dinucleotide (NADH), which may contribute to increased reactive oxygen species (ROS) production via NADH oxidase. This cascade of events, including sorbitol accumulation and oxidative stress, is implicated in the pathogenesis of diabetic complications (Tang et al., 2012). The significance of oxidative stress in diabetic complications is highlighted by increased levels of oxidized DNA, proteins, and lipids, which have been extensively studied (Wiernsperger, 2003). Targeting the polyol pathway, specifically by inhibiting AR using aldose reductase inhibitors (ARIs), has emerged as a potential therapeutic strategy for managing diabetic complications (Julius and Hopper, 2019). Clinical studies have demonstrated that AR inhibitors such as sorbinil, tolrestat, and zopolrestat reduce the occurrence of various diabetic complications, including atherothrombotic cardiovascular disease, myocardial ischemia, retinopathy, nephropathy, and neuropathy (Hotta, 2010; Gamal and Munusamy, 2017; Chang et al., 2019; Gopal et al., 2023). However, despite promising preclinical data, the clinical effectiveness of ARIs remains uncertain, and concerns persist about adverse effects such as hepatic damage and neuropathy (Hotta, 2010).

Natural compounds derived from plants, microorganisms, and marine organisms have shown diverse biological activities and unique chemical structures, making them a promising basis for developing new therapeutics (Mishra and Tiwari, 2011). The advancement of genomics, transcriptomics, and proteomics has been crucial in studying biomarkers and genes related to the development of complex traits (Wesonga and Awe, 2022; Nzungize et al., 2022; Chikwambi et al., 2023; El Abed et al., 2023; Nyamari et al., 2023; Ogbodo et al., 2023; Omar et al., 2024; Alaya et al., 2024; Aribi et al., 2024). Natural products have long been an important source of potential drugs for various diseases and conditions. Approximately 35% of modern medicines are estimated to be derived from natural products. Examples include the anti-cancer drug Taxol from the Pacific yew tree and the anti-malarial drug artemisinin from the Artemisia annua plant (Calixto, 2019). Bioinformatics and *in silico* approaches have experienced huge developments, thereby enabling their applications in various fields such as in identifying potential lead compounds (Enejoh et al., 2025; Hanson et al., 2024), comparative genomics (Awe et al., 2023; Mwanga et al., 2023; Obura et al., 2022) and pipeline development (Ather et al., 2018; Die et al., 2019) and protein structure prediction (Pawar et al., 2024). Natural compounds from Africa present a rich resource for discovering active pharmaceutical ingredients. For example, metformin, a widely used oral antidiabetic drug, originates from *Galegaofficinalis*, a plant traditionally used across North Africa, the Middle East, and Europe to alleviate diabetes symptoms. The discovery of galegine, an active compound extracted from *Galegaofficinalis*, led to the development of metformin as an active ingredient (Witters, 2001; Brasileira De Farmacognosia et al., 2006).

Building on the understanding of aldose reductase’s role in diabetic mechanisms and the potential of natural compounds as therapeutic agents, this study utilized computer-aided drug design methods to identify potential inhibitors. These techniques are valuable tools in pharmaceutical research and allow for cost-effective identification and optimization of potential drug candidates, bypassing the limitations of traditional laboratory-based approaches (Zuhri et al., 2022). We employed a virtual screening approach to screen a database of African natural compounds against aldose reductase to identify the most appropriate inhibitors that could serve as potential therapeutics to treat diabetes and its consequences. The molecular docking approach enabled us to assess the binding affinity and interactions between the selected compounds and the enzyme. To validate the reliability of these interactions, molecular dynamics simulations were performed to provide insights into the stability of the protein-ligand complex over time.

## 2 Methods

**FIGURE 1:**
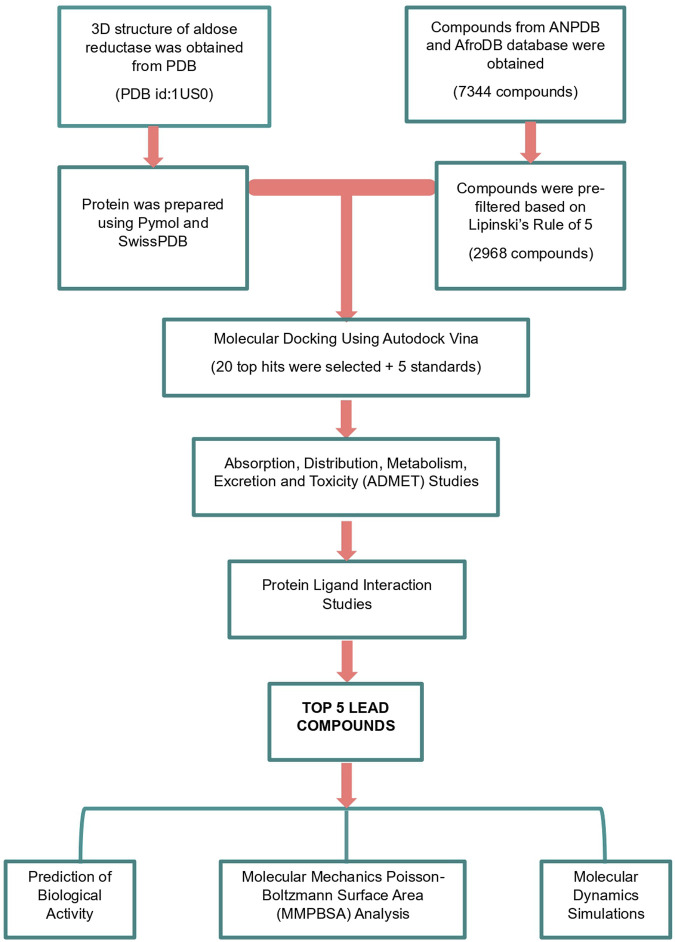
A graphic illustration of the study’s methodology. The methods involved pre-filtering ligands using Lipinski’s rule of five. Molecular docking, pharmacokinetics profiling by assessing the absorption, distribution, metabolism, excretion, and toxicity (ADMET), protein-ligand interaction analysis, and biological activity predictions were used to identify promising leads.

This study employed a systematic methodology to identify potent aldose reductase (AR) inhibitors using computational techniques (Figure 1). The process begins with the preparation of the AR protein and a diverse compound library sourced from AfroDB, NANPDB, and EANPDB. The docking protocol was validated by redocking the experimental ligand and receiver operating characteristic (ROC) curve analysis. Virtual screening was conducted via molecular docking to identify compounds with significant interaction potential for inhibiting AR. Following the initial screening, selected compounds underwent pharmacological evaluation, including ADMET (Absorption, Distribution, Metabolism, Excretion, and Toxicity) predictions and analysis of protein-ligand interactions to ensure favorable drug-like properties. To further investigate the interactions, molecular dynamics simulations, and MM-PBSA calculations are performed to assess the stability and binding free energies of the selected complexes.

### 2.1 Preparation of the aldose reductase protein

The three-dimensional (3D) x-ray diffraction structure of the human AR protein was retrieved from the Research Collaboratory for Structural Bioinformatics (RCSB) Protein Data Bank (PDB) (https://www.rcsb.org/structure/1us0; PDB ID: 1US0) (Berman et al., 2000). The 1US0 structure resolved at 0.66 Å was co-crystalized with its cofactor, the Nicotinamide-adenine dinucleotide phosphate (NADP+), and the inhibitor IDD 594 (Howard et al., 2004). The existing inhibitor and water molecules were removed from the protein’s structure using the PyMOL version 3.0.0 software (DeLano, 2002) and saved in the. pdb format. The SwissPDB viewer (Guex et al., 2009) was used to check and resolve the missing residues. Energy minimization was carried out using GROMACS version 2024 (Abraham et al., 2015), and the output. gro file was converted to a PDB file using PyMOL.

### 2.2 Preparation of compound library

The compounds used for this study were retrieved from the AfroDB Database (Ntie-Kang et al., 2013), the Northern African Natural Products Database (NANPDB), and the East African Natural Product Database (EANPDB) (Ntie-Kang et al., 2017). The AfroDB is a library of natural products containing diverse and highly potent molecules from African medicinal plants. The NANPDB and EANPDB are databases comprising natural products from Northern African and Eastern African sources, respectively. A total of 7,344 compounds obtained from combining the databases were pre-filtered based on Lipinski’s rule of five (Lipinski, 2004) using the Data Warrior software (v.06.01.00) (Sander et al., 2015). Lipinski’s rule of five includes a molecular weight of approximately 500 Da, a partition coefficient (cLogP) of less than five, and the ability to form hydrogen bonds (with no more than five hydrogen bond donors and ten hydrogen bond acceptors). Five standard aldose reductase inhibitors (ARIs), namely, zopolrestat, epalrestat, IDD594, tolrestat, and sorbinil were obtained from the chemistry database PubChem (https://pubchem.ncbi.nlm.nih.gov) and incorporated into the pre-filtered compound libraries.

### 2.3 Validation of docking protocol

#### 2.3.1 Superimposition of co-crystallized protein structure with re-docked complex

In order to validate the docking protocol, the ligand IDD594 was extracted from the co-crystallized structure of aldose reductase obtained from PDB (1US0) and re-docked into the binding site using Autodock Vina software. The docked binding pose of the IDD594 ligand was then superimposed with the experimentally determined pose of the co-crystallized structure by LigPlot+ (v2.2) (Laskowski and Swindells, 2011) and PyMOL.

#### 2.3.2 Receiver operating characteristics (ROC) curve analysis

To further validate the docking protocol,250 decoys of five aldose reductase inhibitors were obtained from the Directory of Useful Decoys and enhanced (DUD-E) web server to generate the ROC curve (Mysinger et al., 2012). Decoys and compounds have similar physicochemical properties but different 2D topologies to the selected inhibitors. The inhibitors comprised mycretin, tolrestat, IDD594, epalrestat, and sorbinil. The area under the curve (AUC) for the ROC curve was generated by screening a total of 250 decoys and five inhibitors against AR using easyROC version 1.3 (Goksuluk et al., 2016).

### 2.4 Virtual screening of the compound libraries

Virtual screening of the pre-filtered compounds and the standard ARIs was performed using the Autodock Vina interface via PyRx software v0.8 (Trott and Olson, 2010; Dallakyan and Olson, 2015). The pre-filtered compounds were obtained as 3D data files (sdf) and then uploaded to PyRx’s Open Babel Converter (O’Boyle et al., 2011). The compounds were energy minimized using the default parameters of the Universal Force Field (UFF) and conjugate gradients for the optimization procedure, which consisted of 200 steps. The. sdf files were then converted to protein data bank partial charge and atom type (.pdbqt) files using Open Babel Converter. The energy-minimized AR protein in. pdb format was also imported into PyRx and converted to. pdbqt. A grid box with dimensions of X = 29.3 Å, Y = 25.1 Å, and Z = 28.4 Å, centered at coordinates X = 40.03 Å, Y = 35.18 Å, and Z = 35.97 Å was used to cover the binding site precisely. The exhaustiveness parameter was maintained at its default value of 8 to ensure efficient docking. The protein was kept in a rigid conformation during the docking process while the ligands were treated as flexible entities during the docking simulations, allowing AutoDock Vina to generate up to 9 conformers for each compound. Five standard AR inhibitors namely, epalrestat, IDD594, sorbinil, tolrestat, and zopolrestat were docked against the AR protein to serve as a benchmark. After virtual screening, compounds that performed better than the standard inhibitors were chosen for further investigation.

### 2.5 Absorption, distribution, metabolism, excretion, and toxicity (ADMET) prediction

SwissADME (Daina et al., 2017) and AdmetSAR (Cheng et al., 2012a; Yang et al., 2019) tools were used for Absorption, Distribution, Metabolism, Excretion, and Toxicity (ADMET) predictions. Ligands in SMILES format were used to generate pharmacological profiles. SwissADME and AdmetSAR provided access to parameters and predictive models for the computation of pharmacokinetics, physicochemical properties, drug-likeness, and toxicity of the preselected compounds.

### 2.6 Protein-ligand interaction

The hydrogen and hydrophobic interactions between AR and the molecules that passed the ADMET test were assessed by LigPlot + using default settings. The protein-ligand complexes generated by PyMOL were saved as. pdb and loaded into Ligplot + to generate 2D schematic representations of the structures and their interactions.

### 2.7 Prediction of biological activity and structural similarity

The Prediction of Activity Spectra for Substances (PASS) (Filimonov et al., 2014) was employed to predict the biological activities of the selected compounds based on Bayesian models. PASS uses a training set of 26,000 compounds with known activities to generate the probability of activity (Pa) and inactivity (Pi) for each compound on a scale from 0.000 to 1.000 (Parasuraman, 2011; Agyapong et al., 2021). A compound with Pa > Pi is considered to have a higher likelihood of the predicted activity. The biological activities assessed in this study were aldose reductase inhibition, antidiabetic, anti-inflammatory, and antioxidant properties. Additionally, structural similarity analysis was conducted using the DrugBank tool (Knox et al., 2024), which provides comprehensive drug data, including over 7,800 drugs, to identify compounds with structural similarities to FDA-approved and experimental drugs. This analysis helped determine whether the selected compounds share common structural features with known bioactive molecules, suggesting potential pharmacological properties.

### 2.8 Molecular dynamics simulation

Molecular dynamics (MD) simulations were conducted for 100 nanoseconds (ns), employing the CHARMM36 all-atom force field (July 2022) and the CHARMM-modified three-point transferable intermolecular potential (TIP3P) water model, within the GROMACS software platform, version 2024 (Abraham et al., 2015). The simulations were executed on the high-performance computing (HPC) infrastructure hosted at the West African Centre for Cell Biology of Infectious Pathogens (WACCBIP), at the University of Ghana. The initial MD simulation focused on the unbound aldose reductase protein, utilizing the aldose reductase raw coordinate file obtained from the Protein Data Bank (PDB) as the starting configuration. Subsequently, MD simulations were conducted for the five docked complexes of aldose reductase. Each simulation employed a dodecahedron box with dimensions of 1.0 nm and was solvated with the SPC water model, which was pre-neutralized. Topology files of the compounds were generated using the CHARMM General Force Field (CGenFF) (Vanommeslaeghe and MacKerell, 2012). Energy minimization was performed over 1,000 steps using the Steepest Descent (SD) algorithm. Position restraints were applied to the AR protein and the ligands, followed by temperature equilibration at 300 K and pressure equilibration at 1 bar, each conducted for 50,000 picoseconds. Subsequently, production MD runs were carried out for 100 nanoseconds, with temperature and pressure maintained at 300 K and 1 bar, respectively. After MD, the output files were visualized and analyzed for the radius of gyration (Rg), root mean square deviations (RMSD), and root mean square fluctuations (RMSF) of the atoms for each amino acid residue using XMGRACE, Version 5.1.19 (Turner, 2005).

### 2.9 Molecular mechanics poisson-boltzmann surface area (MM-PBSA) calculations

The Molecular Mechanics Poisson-Boltzmann Surface Area (MM-PBSA) technique was utilized to compute the binding free energies of the protein-ligand complexes (Wang et al., 2017). Molecular mechanics and continuum solvent models are combined in MM-PBSA to yield the binding energy components and the individual energy contributions of the AR residues. The graphs from the MM-PBSA computations were then plotted using the RStudio programming software version 2023.6.0.421 (Posit team, 2023).

## 3 Results

### 3.1 Preparation of AR protein

In this study, we considered the aldose reductase (AR) protein structure 1US0 with the lowest structure resolution of 0.66 Å and an R-value of 0.094 compared with other AR proteins in the protein database. The selected AR structure was compared with equally solved structures such as 4IGS, 1PWM, and 4LBS, with resolutions 0.85, 0.92, and 0.76 Å, respectively, and R-values of 0.143, 0.129, and 0.134, respectively. A highly resolved structure has well-defined substructures and features, including the active site region, bond density, and significant deviations from standard stereochemistry (Howard et al., 2004). The AR protein with PDB ID: 1US0 exists as a 37.4 kDa monomeric protein with 316 amino acids and an active site that is bound to cofactor NADP^+^ and inhibitor IDD594 which were removed to make the active site available for molecular docking of the compounds in this study.

### 3.2 Preparation of compound library

In preparing the EANPDB, NANPD, and AfroDB compound libraries for molecular docking, Lipinski’s rule of five was applied to pre-filter compounds eliminating those that failed to meet Lipinski’s criteria. Lipinski’s rule of five is a widely accepted method for evaluating the drug-likeness of compounds. It combines computational and experimental techniques to assess the likelihood of absorption or permeation of the compound (Mahgoub et al., 2022). The rule states that a compound is more likely to be a viable drug candidate if it satisfies certain criteria, which include having less than five hydrogen bond donors, less than 10 hydrogen bond acceptors, a molecular weight greater than 500, and a calculated Log P (cLogP) value exceeding five (or MlogP > 4.15) (Lipinski, 2004; Devadasu et al., 2018). After pre-filtering, 2,968 out of 7,344 compounds in compound libraries satisfied the rule and were therefore considered for docking.

### 3.3 Validation of the docking protocol

Validation of a docking protocol is a crucial step in any *in silico* study that utilizes molecular docking. Several studies have shown that the validation process is useful for assessing the accuracy and robustness of a chosen protocol for the specific system being studied (Shivanika et al., 2022; Granchi et al., 2015). Validation is generally achieved using various procedures some of which include, re-docking, super-imposition, use of docking decoys, comparison with other docking programs, and so on (Hevener et al., 2009). However, for this study, validation by superimposition and validation by ROC curve analysis using docking decoys were used to assess the docking protocol.

#### 3.3.1 Validation by superimposition

**FIGURE 2:**
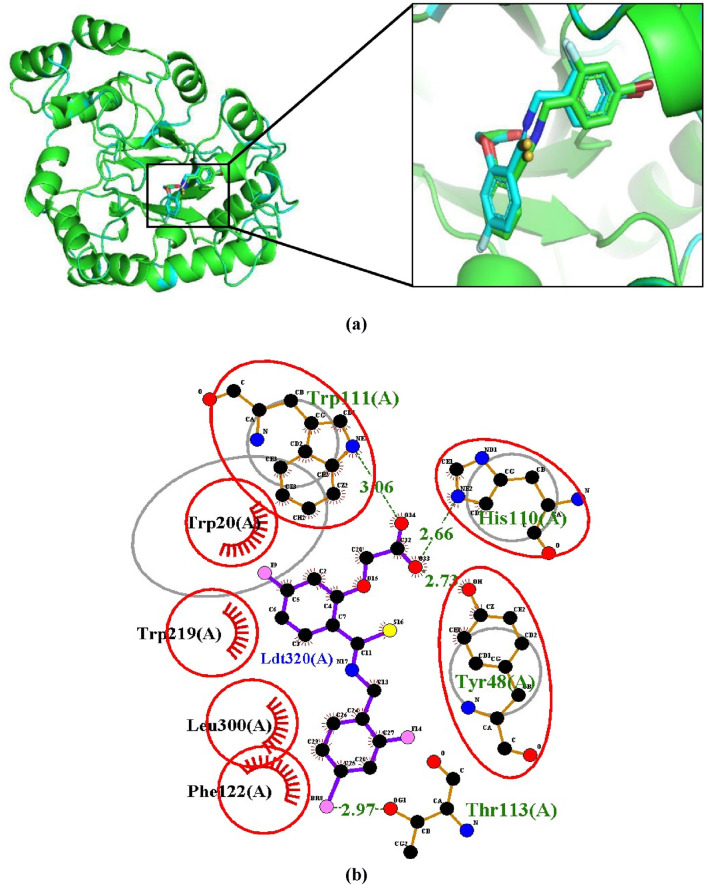
Validation of docking protocol by superimposition **(A)** Superimposed image showing the structural alignment of the docked complex of native IDD594 ligand (green) and re-docked ligand (blue) with protein represented in cartoon and ligand represented in sticks. **(B)** LigPlot+ of superimposition between the co-crystallized ligand of aldose reductase (1US0) and the re-docked IDD594 ligand. Red circles represent the superimposed molecular interactions between the co-crystallized and the re-docked ligands.

This validation method involves extracting the inhibitor (IDD594) from the experimental crystal (1US0) and re-docking it into the binding site of the aldose reductase protein. The re-docked structure was superimposed using PyMOL (v.3.0.0) on the co-crystallized protein-ligand complex resulting in an RMSD of 0.211Å (Figure 2A). Using LigPlot (v.2.2.8), IDD594 was observed to bind into the binding site via hydrogen bonding with His110, Trp111, Tyr48, Thr113 and hydrophobic interactions with Trp20, Trp219, Phe122, and Leu300 as shown in Figure 2B. The validation process with an RMSD of 0.000 is indicative of a very high degree of similarity between the docked ligand pose and the reference ligand pose (Hevener et al., 2009), thereby suggesting a high degree of accuracy in predicting the ligand’s binding pose with similarity in hydrogen and hydrophobic bond interactions.

#### 3.3.2 Validation by ROC curve analysis

**FIGURE 3:**
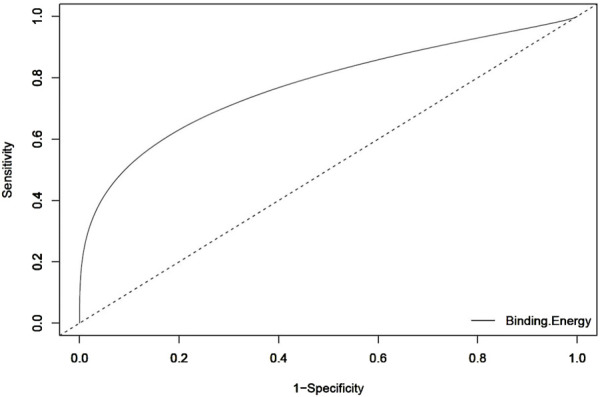
An ROC curve generated after screening 255 compounds consisting of five inhibitors and 250 decoys against the 3D model of the aldose reductase. An acceptable AUC of 0.773 was obtained.

To further validate the docking protocol used in the study, the Area under the curve (AUC) of the Receiver Operating Characteristic (ROC) curve was used. This is an indicator for assessing a docking model’s capacity to differentiate between docked decoys and active ligands (Bowers and Zhou, 2019). When tested against the aldose reductase protein (1US0), the ROC curve depicts the overall docking performance in distinguishing between active and decoy ligands (Figure 3). An AUC of ROC closer to 1 indicates that the model can differentiate between active ligands and decoys more effectively (Guterres and Im, 2020). The results showed an AUC value of 0.773 for the ROC curve when evaluating the 5 active inhibitors and 250 decoys independently against the aldose reductase model 1US0.

### 3.4 Molecular docking of compounds

Molecular docking is a method for predicting the binding mode and binding affinity of a small molecule compound to the binding site of a target protein based on its structural properties (Guterres and Im, 2020; Khan et al., 2023). The binding affinities for the 2,968 pre-filtered compounds ranged from −12.3 to −3.6 kcal/mol. The more negative the affinity score, the stronger the bond between the compound and the protein. Zopolrestat, a standard inhibitor, had the highest binding affinity of −9.9 kcal/mol and was used as the benchmark for selecting the best compounds. This resulted in the selection of 105 compounds (Supplementary Table S1). However, due to computational limitations, only the best 20 compounds were selected for downstream analysis. The molecular docking results for the top 20 compounds and 5 standard aldose reductase inhibitors are shown in Table 1. The affinity scores of these 20 compounds ranged between −12.3 kcal/mol and −10.7 kcal/mol. The compound 4,5-di-p-trans-coumaroylquinic_acid had the highest binding affinity score of −12.3 kcal/mol, while 1,6-di-o-p-hydroxybenzoyl-beta-d-glucopyranoside had the lowest binding affinity score of −10.7 kcal/mol. All top 20 compounds had a higher binding affinity than the known standard inhibitors, with binding affinity ranging between −9.9 kcal/mol and −7.7 kcal/mol.

**TABLE 1:** The top 20 compounds and standard inhibitors selected after molecular docking.

Compound name	Binding affinity (kcal/mol)
Top 20 compounds (AfroDB/EANPDB/NANPDB)	
4,5-di-p-trans-coumaroylquinic_acid	−12.3
(+)-pipoxide	−11.4
Thymelol	−11.4
Zinc000095485961	−11.2
Rutamontine	−11.1
(−)-tingtanoxide	−11.0
Tricoccin_s13_acetate	−11.0
Lactupicrin	−11.0
Naamidine A	−11.0
Zinc000000134782	−10.9
Sigmoidin-b-4′-methylether diacetate	−10.9
(−)-pipoxide	−10.9
Abyssinone_ii	−10.8
(+)-strigol	−10.8
Norisojamicin	−10.8
Calopogonium_isoflavone_b	−10.8
Isosamarcandin	−10.8
(+)-pipoxide-2-methyl_ether	−10.8
Zinc000095485890	−10.8
1,6-di-o-p-hydroxybenzoyl-beta-d-glucopyranoside	−10.7
Standard inhibitors	
Epalrestat	−8.8
IDD594	−8.1
Sorbinil	−7.4
Tolrestat	−7.6
Zopolrestat	−9.9

### 3.5 Absorption, distribution, metabolism, excretion, and toxicity (ADMET) prediction

ADMET predictions were performed on the top 20 compounds to assess their drug-like properties. A potential lead compound should have a favorable ADMET profile, including appropriate absorption, distribution, metabolism, excretion, and toxicity properties. The results from SwissADME describe the absorption, distribution, and metabolism of the ligands in this study which are illustrated in Tables 2, 3.

**TABLE 2:** Drug-likeness and water solubility of the top 20 compounds and standard inhibitors.

Ligands	MW	#HA	#HD	Log P	Log S	Solubility	LV	Bio Sc
4,5-di-p-trans-coumaroylquinic acid	484.45	10	5	1.98	−3.91	Soluble	0	0.11
(+)-pipoxide	366.36	6	1	2.76	−3.56	Soluble	0	0.55
Thymelol	352.29	7	1	2.87	−4.44	Moderately soluble	0	0.55
Zinc000095485961	446.45	9	5	2.08	−3.11	Soluble	0	0.55
Rutamontine	352.29	7	1	2.87	−4.44	Moderately soluble	0	0.55
(−)-tingtanoxide	408.4	7	0	3.95	−4.02	Moderately soluble	0	0.55
Tricoccin s13 acetate	470.6	6	0	3.34	−5.62	Moderately soluble	0	0.55
Lactupicrin	410.42	7	2	1.99	−2.9	Soluble	0	0.55
Naamidine A	433.46	6	2	2.44	−4.27	Moderately soluble	0	0.55
Zinc000000134782	344.36	4	0	3.53	−5.29	Moderately soluble	0	0.55
Sigmoidin-b-4′-methylether diacetate	454.47	8	1	3.97	−5.23	Moderately soluble	0	0.55
(−)-pipoxide	366.36	6	1	3.25	−3.56	Soluble	0	0.55
Abyssinone_ii	324.37	4	2	2.83	−4.68	Moderately soluble	0	0.55
(+)-strigol	346.37	6	1	2.89	−2.65	Soluble	0	0.56
Norisojamicin	364.35	6	1	3.51	−4.71	Moderately soluble	0	0.55
Calopogonium_isoflavone_b	348.35	5	0	3.64	−4.86	Moderately soluble	0	0.55
Isosamarcandin	400.51	5	2	3.47	−5	Moderately soluble	0	0.55
(+)-pipoxide-2-methyl_ether	380.39	6	0	3.08	−3.91	Soluble	0	0.55
Zinc000095485890	438.47	7	0	3.01	−3.64	Soluble	0	0.55
1,6-di-o-p-hydroxybenzoyl-beta-d-glucopyranoside	420.37	10	5	1.25	−2.66	Soluble	0	0.55
Standard Inhibitors								
Epalrestat	319.4	4	1	2.73	−2.77	Soluble	0	0.55
Zopolrestat	419.38	8	1	2.66	−4.83	Moderately soluble	0	0.56
Sorbinil	236.2	4	2	1.39	−2.06	Soluble	0	0.55
Tolrestat	357.35	6	1	2.42	−4.29	Moderately soluble	0	0.56
IDD49	416.24	5	2	2.4	−4.69	Moderately soluble	0	0.56

where # HA, Number of hydrogen bond acceptors; # MW, Molecular weight; # HD, Number of hydrogen bond donors; # Bio Sc, Bioavailability Score; # LV, Lipinski’s rule violations.

**TABLE 3:** Pharmacokinetics properties of the predicted compounds and standard inhibitors. The pharmacokinetics properties comprised cytochrome inhibition, the blood-brain barrier permeability (BBB), P-glycoprotein (P-gp) substrates, and gastrointestinal (GI) absorption and log Kp.

Ligands	GI absorption	BBB permeability	Pgp substrate	CYP1A2 inhibitor	CYP2C19 inhibitor	CYP2C9 inhibitor	CYP2D6 inhibitor	CYP3A4 inhibitor	log Kp (cm/s)
4,5-di-p-trans-coumaroylquinic acid	Low	No	Yes	No	No	No	No	No	−7.67
(+)-pipoxide	High	No	No	Yes	No	Yes	No	Yes	−6.75
Thymelol	High	No	No	Yes	No	Yes	No	No	−6.14
Zinc000095485961	Low	No	Yes	No	No	No	No	No	−8.1
Rutamontine	High	No	No	Yes	No	Yes	No	No	−6.14
(−)-tingtanoxide	High	No	No	No	Yes	Yes	No	Yes	−6.6
Tricoccin_s13_acetate	High	No	Yes	No	No	Yes	No	No	−5.73
Lactupicrin	High	No	Yes	No	No	No	No	No	−8.02
Naamidine A	High	No	Yes	No	Yes	Yes	No	Yes	−6.91
Zinc000000134782	High	Yes	No	Yes	Yes	Yes	No	Yes	−5.2
Sigmoidin b 4′-methylether diacetate	High	No	No	Yes	Yes	Yes	No	Yes	−5.88
(−)-pipoxide	High	No	No	Yes	No	Yes	Yes	Yes	−6.75
Abyssinone_ii	High	Yes	No	Yes	Yes	Yes	Yes	Yes	−5.28
(+)-strigol	High	No	No	No	No	No	No	No	−7.52
Norisojamicin	High	No	No	Yes	Yes	Yes	No	Yes	−5.99
Calopogonium_isoflavone_b	High	Yes	No	Yes	Yes	Yes	No	Yes	−5.64
Isosamarcandin	High	No	Yes	No	No	No	Yes	Yes	−5.79
(+)-pipoxide-2-methyl_ether	High	Yes	No	Yes	Yes	Yes	Yes	Yes	−6.45
Zinc000095485890	High	No	No	No	No	No	No	No	−7.58
1,6-di-o-p-hydroxybenzoyl-beta-d-glucopyranoside	Low	No	Yes	No	No	No	No	No	−8.43
Standard Inhibitors									
Epalrestat	High	No	Yes	Yes	Yes	No	No	Yes	−3.79
Zopolrestat	High	No	No	No	Yes	Yes	No	No	−6.34
Sorbinil	High	No	Yes	No	No	No	No	No	−7.19
Tolrestat	High	No	No	Yes	Yes	No	No	No	−5.87
IDD49	High	No	No	Yes	Yes	Yes	Yes	No	−6.18

All the compounds including the standard inhibitors complied with Lipinski’s rule of five as shown in Table 2, because the compound library was prefiltered based on Lipinski’s rule of 5. The bioavailability score is the likelihood that a substance will possess an oral bioavailability of no less than 10% in rats and be detected by Caco-2 permeability (Stelzl et al., 2005). This is represented by a value range of 0–1 in SwissADME, which can be translated to a percentage (Martin, 2005). All the ligands in this research study have a bioavailability score of 0.55 except 4,5-di-p-trans-coumaroylquinic acid, which has a bioavailability score of 0.11, and (+)-strigol has 0.56; this could be translated to a bioavailability score of 55%, 11%, and 56%, respectively (Table 2). However, all the standards had a bioavailability score of 0.55 except for epalrestat and sorbinil which had a score of 0.56.

As indicated in Table 3, compounds that were not inhibitors of any cytochrome P450 enzymes include 4,5-di-p-trans-coumaroylquinic acid, Zinc000095485961, Lactupicrin, (+)-strigol, and Zinc000095485890**.** These results suggest that they are less likely to be involved in drug-drug interactions and have adequate drug elimination properties via metabolic biotransformation. All the predicted ligands exhibited high gastrointestinal absorption except 4,5-di-p-trans-coumaroylquinic acid. Zinc000000134782, Abyssinone II, and (+)-pipoxide-2-methyl ether compounds were found to show potential permeability through the blood-brain barrier among all the compounds from the SwissADME prediction. *In vivo* and *in vitro* studies have shown that P-glycoprotein is important for drug absorption and clearance in the liver and kidney. In the brain, it also acts as a rate-limiting factor for drug uptake from blood circulation into the brain; in the intestinal lumen, it is a binding agent for drug absorption into epithelial cells (Lin and Yamazaki, 2003). Drugs that bind to P-glycoprotein (Pgp) are regarded as Pgp substrates. 4,5-di-p-trans-coumaroylquinic acid, Zinc000095485961, Tricoccin_s13_acetate, Lactupicrin, Naamidine A, Isosamarcandin, and 1,6-di-o-p-hydroxybenzoyl-beta-d-glucopyranoside show potential to bind to P-glycoprotein from the SwissADME prediction. Log Kp (expressed in cm/s) represents a crucial indicator of a drug or ligand’s ability to permeate the skin, especially if the mode of administration is transdermal (Chen et al., 2018). A compound is typically considered to have limited skin permeability when its log Kp is greater than −2.5 cm/s (Pires et al., 2015). In the study, all the predicted compounds have values ranging from −8.43 to −5.20 cm/s, indicating extremely low skin permeability.

The prediction of the toxicological properties of the ligands used for docking was achieved using admetSAR 2.0 (Cheng et al., 2012b). Hepatotoxicity, acute oral toxicity, nephrotoxicity, carcinogenicity, and mutagenicity toxicological properties were recorded, as shown in Table 4. The results from the admetSAR also showed that none of the ligands is carcinogenic (Table 4). The compounds that exhibited positive toxicology predictions for hepatotoxicity, acute oral toxicity, nephrotoxicity, carcinogenicity, and mutagenesis were eliminated leaving eight compounds namely, (+)-pipoxide, Zinc000095485961, Naamidine A, Sigmoidin B 4′-methylether diacetate, (−)-pipoxide, (+)-strigol, Isosamarcandin, and 1,6-di-O-p-hydroxybenzoyl-beta-D-glucopyranoside. These selected compounds stand out due to their high bioavailability, minimal Central Nervous System (CNS) side effects, low drug-drug interaction potential, and excellent safety profiles across key toxicological endpoints, making them promising candidates for targeting aldose reductase in managing diabetic complications. Additionally, epalrestat and IDD549, where the standard inhibitors showed no toxicity for the selected toxicity profiles.

**TABLE 4:** The toxicology of the ligands is based on the linear regression model prediction from admetSAR. The negative symbol (−) indicates a negative prediction of being toxic while the positive symbol (+) indicates a negative prediction of being non-toxic.

Ligands	Hepatotoxicity	Acute oral toxicity	Nephrotoxicity	Carcinogenicity	Ames mutagenesis
4,5-di-p-trans-coumaroylquinic acid	**+**	−	−	−	−
(+)-Pipoxide	−	−	**-**	−	−
Thymelol	−	−	**+**	−	−
Zinc000095485961	−	−	**-**	−	−
Rutamontine	−	−	**+**	−	−
(−)-Tingtanoxide	−	−	**+**	−	−
Tricoccin S13 Acetate	**-**	−	**+**	−	−
Lactupicrin	**+**	−	**+**	−	−
Naamidine A	−	−	**-**	−	−
Zinc000000134782	−	−	**+**	−	**+**
Sigmoidin b 4′-methylether diacetate	−	−	−	−	−
(−)-Pipoxide	−	−	−	−	−
Abyssinone_ii	**+**	−	**+**	**-**	−
(+)-Strigol	−	−	**-**	−	−
Norisojamicin	**+**	−	**+**	−	−
Calopogonium Isoflavone B	**+**	−	**+**	−	−
Isosamarcandin	−	−	−	−	−
(+)-Pipoxide-2-methyl Ether	**+**	−	−	−	−
Zinc000095485890	**+**	−	**+**	−	−
1,6-di-o-p-hydroxybenzoyl-beta-d-glucopyranoside	−	−	−	−	−
Standard Inhibitors					
Epalrestat	−	−	−	−	−
Zopolrestat	**+**	−	**+**	−	−
Sorbinil	**+**	−	**+**	−	−
Tolrestat	−	**+**	**+**	−	−
IDD49	−	−	−	−	−

### 3.6 Protein-ligand interactions

**FIGURE 4:**
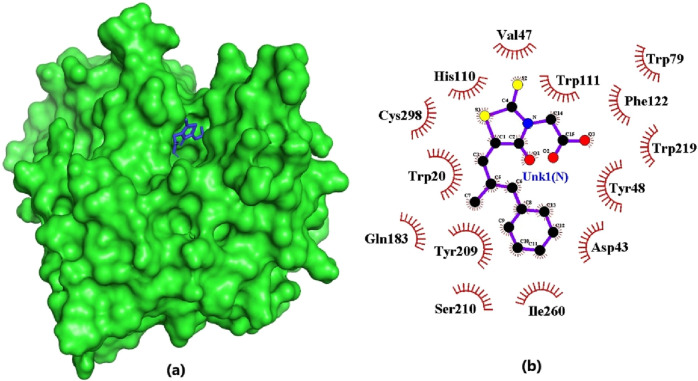
Epalrestat docked firmly in the binding pocket and interacting with critical residues. **(A)** Surface view of the AR protein and epalrestat (blue). **(B)** 2D diagram of the protein-ligand interaction generated using LigPlot+.The ligand is colored in purple, and hydrophobic contacts are represented as red spoke arcs.

The eight compounds and the standard inhibitor (epalrestat) identified through the ADMET studies underwent analysis to assess their interactions with the active site of the aldose reductase protein (Table 5). The amino acids comprising this active site were determined from literature reviews, as follows: His110, Asp43, Lys77, Cys298, and Tyr48 (Tarle et al., 1993); and Trp111, Trp20, Phe122, Thr113, Leu300, Ser210, and Trp219 (Ashik et al., 2022). Notably, 1,6-di-o-p-hydroxybenzoyl-beta-d-glucopyranoside, (+)-pipoxide, and (+)-strigol exhibited hydrogen bonding interactions with residue Trp111, with bond lengths of 3.03, 3.16, and 3.29, respectively. Naamidine A formed hydrogen bonds with Trp20 and Thr113, with bond lengths of 3.01 and 3.13, respectively. Moreover, 1,6-di-o-p-hydroxybenzoyl-beta-d-glucopyranoside and Zinc000095485961 were observed to interact with Ile260, His110, and Thr113 via hydrogen bonds (Table 5). However, Sigmoidin-b-4′-methylether diacetate and (+)-strigol, despite forming hydrogen bonds, did not interact with the crucial residues of the aldose reductase active site, leading to their exclusion from further studies. Isosamarcandin was also excluded due to its lack of interaction with any residues via hydrogen bonding. Selection of compounds solely based on interactions with only the critical residues of the aldose reductase active site resulted in five compounds being selected as the top compounds. Notably, the standard inhibitor interacted with the active site solely through hydrophobic interactions implying that our selected compounds may form stronger interactions when bound to AR than the standard epalrestat (Table 5; Figure 4).

**TABLE 5:** This table depicts the interactions between AR binding site residues and the top 20 compounds via hydrogen bonding and hydrophobic interactions. The length of the hydrogen bonds’ interactions is also shown.

Compound	Hydrogen bonds	Hydrogen bond length (Å)	Hydrophobic interactions
(+)-pipoxide	His110 Trp111	3.01 3.03	Trp309, Lue300, Thr114, Cys303, Phe122, Phe115, Trp79, Val47, Gln183, Tyr209, Asp43, Tyr48, Ile3260, Ser210, Trp20, Cys298
Zinc000095485961	Trp20 Ile260 His110 Thr113	2.81 2.78 3.03 2.87	Cys303, Phe122, Tyr309, Trp111, Trp79, Leu300, Tyr48, Asp43, Lys262, Ser210, Tyr209, Cys298, Trp219, Phe115
Naamidine A	Trp20 Thr113	3.01 2.93	Ala299, Phe115, Tyr209, Tyr48, Cys298, His110, Trp111, Val47, Trp219, Trp79, Leu300, Cys303, Tyr309
Sigmoidin B 4′-methylether diacetate	Gln183 Ser210 Trp20	2.42 2.83 2.99, 3.14	Lys262, Tyr209, Lys77, His110, Trp111, Phe115, Leu300, Cys298, Phe122, Ala299, Phe122, Trp219, Tyr48, Lys21
(−)-pipoxide	His110 Cys298	2.87, 3.27 3.33	Trp111, Trp79, Phe115, Tyr309, Val 47, Tyr48, Tyr209, Ser210, Asp43, Trp20, Gln183, Phe122, Leu300, Thr113, Cys303
(+)-strigol	Trp111, Asn160	3.16 3.05	Cys298, Tyr48, Phe122, Trp219, Val47, Trp20, Lys262, Gly18, Tyr209, Ser210, His110
Isosamarcandin	-	-	Arg69, Tyr103, Asp105, Arg69, Tyr103, Asp105, Ile58, Leu62, Arg69, Leu72, Lys100, Leu101, Asp102, Tyr103, Lys154
1,6-di-O-p-hydroxybenzoyl-beta-D-glucopyranoside	Thr113 His110 Trp111 Ile260	2.96 3.29 2.78 3.31	Phe122, Phe115, Tyr209, Gln183, Ser210, Trp20, Trp48, Asp43, Leu300, Trp79, Tyr309, Ala299, Cys303
Epalrestat (Standard Inhibitor)	-	-	His110, Val47, Trp111, Phe122, Trp79, Trp219, Tyr48, Asp43, Ile260, Ser210, Tyr209, Gln183, Trp20, Cys298

### 3.7 Prediction of biological activity and structural similarity of the 5 lead compounds

The PASS software predicted the biological activities of the five selected compounds. Zinc000095485961 and 16-di-o-p-hydroxybenzoyl-beta-D-glucopyranoside were predicted to possess all four biological activities which were aldose reductase inhibition, antidiabetic, anti-inflammatory, and antioxidant with high Pa values for each activity (Table 6). Zinc000095485961 had Pa values of 0.679 for antioxidant, 0.636 for anti-inflammatory, 0.596 for antidiabetic, and 0.109 for aldose reductase inhibition, all greater than their Pi values (Table 6). Similarly, 16-di-o-p-hydroxybenzoyl-beta-D-glucopyranoside exhibited Pa values of 0.724 for anti-inflammatory, 0.681 for antioxidant, 0.568 for antidiabetic, and 0.205 for aldose reductase inhibition, also showing strong potential for these activities. (+)-Pipoxide and (−)-Pipoxide were predicted to have anti-inflammatory (Pa = 0.327) and antioxidant (Pa = 0.162) activities, although they were not predicted to have aldose reductase inhibitory or antidiabetic properties. Naamidine A did not exhibit significant activity in any of the assessed biological categories according to the PASS predictions, but its known anticancer properties suggest other potential uses.

**TABLE 6:** The table shows the names of the compounds and their predicted biological activity with their corresponding probability of activity (Pa) and the probability of inactivity (Pi). Selected pharmacological activity in this study includes aldose reductase inhibition, anti-inflammatory, antidiabetic, and antioxidant activity. Additionally, the table shows structural similarity scores for compounds compared to known drugs.

Compounds	Pa	Pi	Pa > Pi	Predicted pharmacologic activity	Structurally similar drug	Similarity score
Zinc000095485961	0.679	0.004	Yes	Antioxidant	Acteoside	0.891
	0.636	0.025	Yes	Anti-inflammatory		
	0.596	0.013	Yes	Antidiabetic	Echinacoside	0.887
	0.109	0.016	Yes	Aldose reductase inhibitor		
1,6-di-o-p-hydroxybenzoyl-beta-d-glucopyranoside	0.724	0.013	Yes	Anti-inflammatory	Beta-1,2,3,4,6-Penta-O-Galloyl-D-Glucopyranose	0.739
	0.681	0.004	Yes	Antioxidant		
	0.568	0.015	Yes	Antidiabetic		
	0.205	0.005	Yes	Aldose reductase inhibitor	Tannic acid	0.873
(+)- Pipoxide	0.327	0.138	Yes	Anti-inflammatory	None	None
	0.162	0.089	Yes	Antioxidant		
- (−) Pipoxide	0.327	0.138	Yes	Anti-inflammatory	None	None
	0.162	0.089	Yes	Antioxidant		
Naamidine	-	-	-	None	None	None
Epalrestat	0.432	0.003	Yes	Aldose reductase inhibitor	None	
	0.407	0.042	Yes	Antidiabetic		
	0.248	0.121	Yes	Anti-Inflammatory		

In the structural similarity analysis (Table 6), Zinc000095485961 showed a high similarity score with Acteoside (0.891) and Echinacoside (0.887), both of which are known bioactive compounds. Also, 16-di-o-p-hydroxybenzoyl-beta-D-glucopyranoside exhibited notable structural similarity to Beta-12346-Penta-O-Galloyl-D-Glucopyranose (0.873) and Tannic Acid (0.739). These findings suggest that these compounds may share pharmacological properties with known drugs. In contrast, (+)-Pipoxide, (−)-Pipoxide, and Naamidine A did not demonstrate significant structural similarity to any known drugs in the DrugBank database.

### 3.8 Molecular dynamics simulation

#### 3.8.1 Root mean square deviation (RMSD)

**FIGURE 5:**
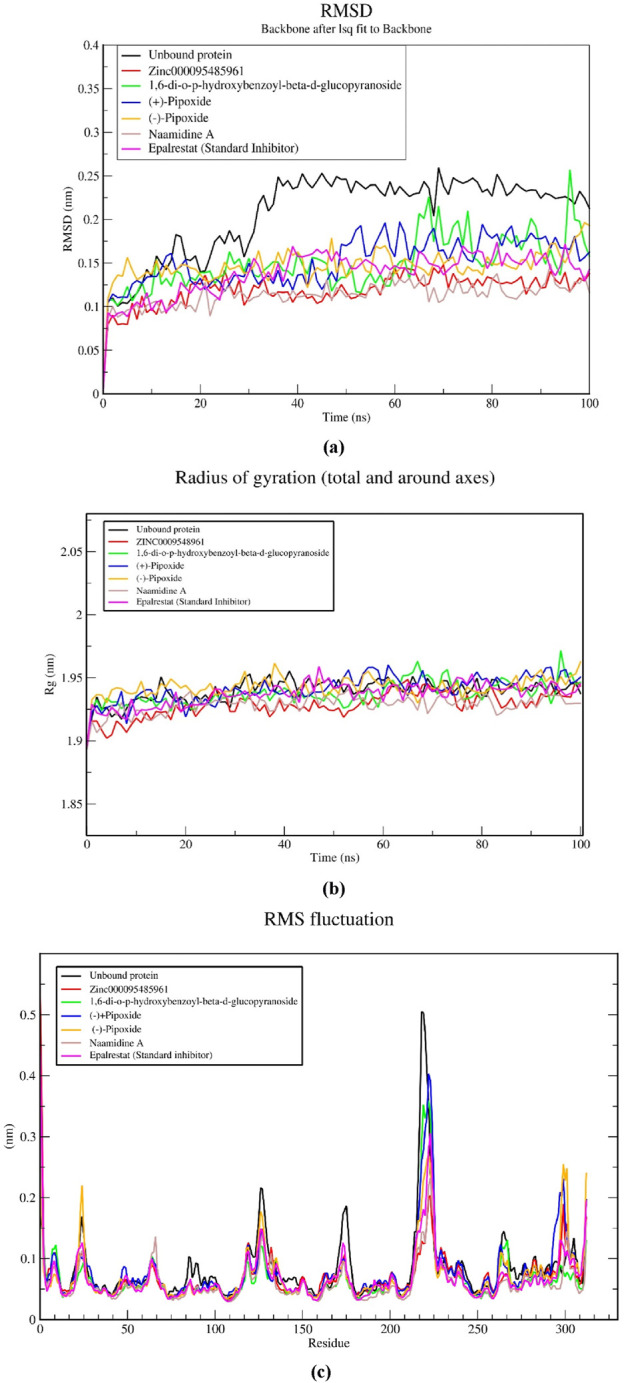
MD simulations on five selected natural compounds and the standard inhibitor (epalrestat). A 100 ns MD simulation was performed to analyze the structural stability and conformational changes of aldose reductase when bound to the complexes. The parameters considered shown in the graphs include **(A)** the root mean square deviation (RMSD), **(B)** the radius of gyration (Rg), and **(C)** the root mean square fluctuation (RMSF).

An RMSD plot over simulation time revealed the backbones of the five complexes after 100 ns in comparison to the unbound AR protein and a standard inhibitor (Epalrestat-AR) complex (Figure 5A). The unbound protein at the initial start of the simulation had an RMSD of 0.11 nm that gradually increased to 0.16 nm and after 16 ns decreased steadily till 22 ns where it attained an RMSD of 0.14 nm. A steady increase was observed after the 22 ns time and maintained a relatively steady RMSD of 0.24 nm at 38 ns till the end of the 100 ns simulation. The standard inhibitor (Epalrestat-AR) complex had an initial RMSD of 0.09 nm which increased steadily to 0.17 nm at 40 ns and then decreased to 0.14 nm at 65 ns after RMSD was maintained at an average RMSD of 0.14 nm in the remaining simulation time. Zinc0009548961-AR complex rose from 0 nm to 0.08 nm at the start of the simulation and then maintained an average RMSD of 0.12 nm over the remaining simulation time. The 1,6-di-o-p-hydroxybenzoyl-beta-d-glucopyranoside (1,6 DHG)-AR complex had an RMSD of 0.11 at the start of the simulation and peaked at 0.15 nm/25 ns; then it decreased to 0.125 at a maintained RMSD. It peaked around 70 ns and later averaged at about 0.12 nm over the remaining simulation time. For (+)- pipoxide, the RMSD started at 0.1 nm and steadily increased to 0.15 nm within the first 12 ns. It then declined to 0.125 nm after 25 ns. The RMSD was maintained at 0.125 nm till 50 ns where it increased to 0.18 nm and then maintained an average RMSD of 0.18 nm till the end of the simulation. (−)- Pipoxide initially had an RMSD of 0.15 nm at the start of the simulation which then declined to 0.13 nm. An average RMSD of 0.13 nm was then maintained throughout the simulation. The Naamidine-AR complex showed the most stable conformation by maintaining an RMSD of 0.1 nm in the 100 ns simulation.

#### 3.8.2 Radius of gyration (Rg)

The compactness of the complexes was evaluated using the radius of gyration (Rg). The Rg values obtained from the simulations showed that all AR-complexes including the unbound protein (Figure 5B) remained in their compact (folded) form throughout 100 ns. All AR complexes maintained their Rg from the beginning of the simulation (0 ns) to the end (100 ns) within or fluctuations. The average Rg of the unbound protein and AR-complexes, epalrestat, (+)-pipoxide, Zinc000095485961, 1,6-di-o-p-hydroxybenzoyl-beta-d-glucopyranoside, Naamidine A and (−)-pipoxide were 1.940, 1.937, 1.929, 1.937, 1.929, 1.941 and 1.943 nm, respectively. The differences in Rg between the AR-complexes and the standard inhibitors were small however, Zinc000095485961, Naamidine A, and 1,6-di-o-p-hydroxybenzoyl-beta-d-glucopyranoside had a slightly lower Rg compared to the unbound protein and epalrestat.

#### 3.8.3 Root mean square fluctuation (RMSF)

The stability of the individual residues was assessed using their Root Mean Square Fluctuation (RMSF) plots (Figure 5C). All the complexes possessed similar residue fluctuations within the same regions, with little deviation from the unbound protein and the epalrestat-AR complex (Figure 5C). High residue fluctuations were observed within amino acid residues at positions 2, 8, 25, 118, 125, 173, 224, 225, 264, and 312. Residue fluctuations between 0.1 and 0.2 nm were generally observed within the residues. However, a rapid increase to 0.5 nm RMSF within residues 205–225 was observed and this might be indicative of the region with the highest flexibility.

### 3.9 MM-PBSA calculations of ligand-receptor complexes

**FIGURE 6:**
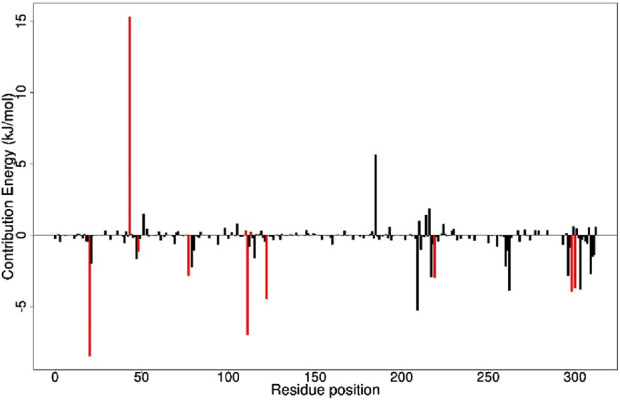
Molecular Mechanics Poisson-Boltzmann Surface Area (MMPBSA) plot of binding free energy contribution per residue of AR- Zinc000095485961 complex. Fluctuations by selected critical residues of AR are shown in red.

The study employed the MM-PBSA approach to elucidate the binding free energies of the top five selected compounds: (+)-pipoxide, Zinc000095485961, Naamidine A, (−)-pipoxide, and 1,6-di-o-p-hydroxybenzoyl-beta-d-glucopyranoside compared to the standard inhibitor (epalrestat). These compounds exhibited binding free energies of −115.073, −106.483, −98.523, −93.751, and −92.007 kJ/mol, respectively Notably, all five compounds displayed higher binding free energy, Van der Waals energy, electrostatic energy, polar solvation energy, and Solvent-Accessible Surface Area (SASA) when compared to epalrestat (Table 7). Additionally, the contribution of each residue’s energy via MM-PBSA decomposition was determined to identify the active site residues of AR involved in ligand binding. Residues with energy contribution exceeding >5.0 or < −5.0 are considered critical residues for protein-ligand binding (Kwofie et al., 2019). For Zinc000095485961 Trp20, Trp111, and Trp219 were the identified critical residues with binding energies of 9.453, 8.032, and 5.031 kJ/mol (Figure 6), respectively. Asp43, a critical residue contributed a high energy of 15.089 kJ/mol in all complexes. For 1,6-di-o-p-hydroxybenzoyl-beta-d-glucopyranoside, Trp20, Trp111, and Leu300 were identified as critical residues with contributed energies of −6.564, −8.187, and 5.342 kJ/mol, respectively (Supplementary Figure S1). Asp43 also had a significantly high energy of 18.011 kJ/mol. Trp208 was not a critical residue but contributed less than −5 kJ/mol. Regarding (+)-pipoxide, Trp20, Trp111, and Phe122 were the critical residues with energies exceeding 5 and below −5 (Supplementary Figure S2). Although Trp43 is not a critical residue, it showed a significant contribution of −5.956 kJ/mol. For (−)- pipoxide-AR complex, Trp20 contributed low energy of −7.654 kJ/mol, with Trp48 also peaking at −9.765 kJ/mol (Supplementary Figure S3). Asp 43 contributed an energy of 9.678 kJ/mol, although it is not a critical residue. Finally, in the naamidine-AR complex, Trp20, Trp111, and Leu300 were the critical residues with a low binding affinity of −6.987, −7.865, and −6.132 kJ/mol, respectively (Supplementary Figure S4). Chemical structures for the five leads and epalrestat are shown in Table 8.

**TABLE 7:** The table below shows the binding energies and the contributing energy terms of the AR-ligand complexes from the MMPBSA calculation. The values are presented in average ± standard deviations in kJ/mol.

Ligands	van der Waal energy (kJ/mol)	Electrostatic energy (kJ/mol)	Polar solvation energy (kJ/mol)	SASA energy (kJ/mol)	Binding energy (kJ/mol)
Zinc000095485961	−222.393 ± 4.184	−59.714 ± 1.794	190.605 ± 3.480	−23.641 ± 0.441	−115.073 ± 3.158
1,6-di-o-p-hydroxybenzoyl-beta-d-glucopyranoside	−227.950 ± 1.075	−42.232 ± 1.401	185.543 ± 1.427	−21.900 ± 0.082	−106.483 ± 1.452
(+)-pipoxide	−174.413 ± 1.379	−34.938 ± 1.340	130.427 ± 1.988	−19.550 ± 0.115	−98.523 ± 1.465
(−)-pipoxide	−161.391 ± 2.271	−18.979 ± 0.806	105.061 ± 1.682	−18.441 ± 0.225	−93.751 ± 1.871
Naamidine A	−177.856 ± 2.899	−32.891 ± 2.020	138.645 ± 2.783	−19.828 ± 0.171	−92.007 ± 2.857
Epalrestat	−116.058 ± 5.800	−26.939 ± 1.639	92.795 ± 4.964	−12.995 ± 0.624	−63.471 ± 4.737

**TABLE 8:** Chemical structures of (A–E) the top 5 identified compounds and (F) Epalrestat (Standard Inhibitor).

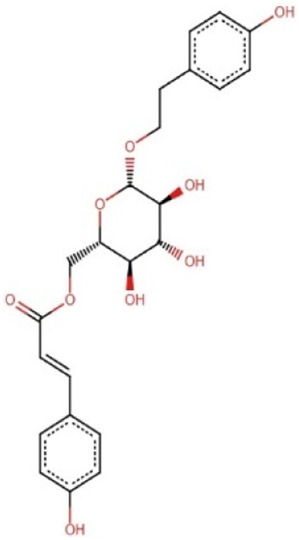 A Zinc000095485961	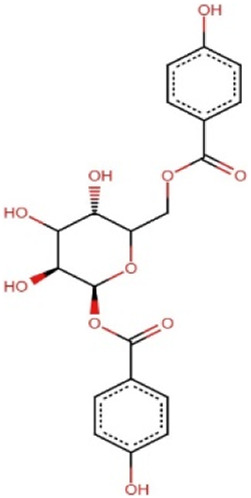 B 1,6-di-o-p-hydroxybenzoyl-beta-d-glucopyranoside	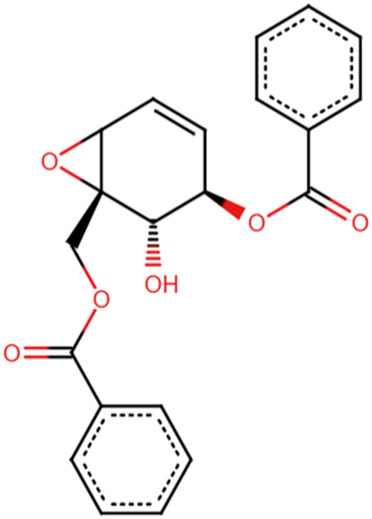 C (+)-Pipoxide
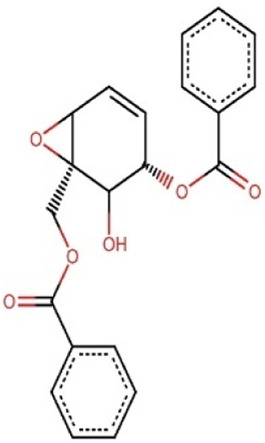 D (−)-Pipoxide	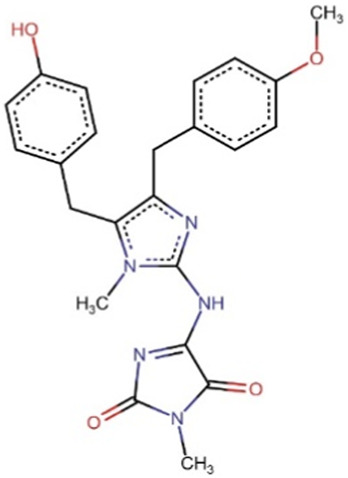 E Naamidine A	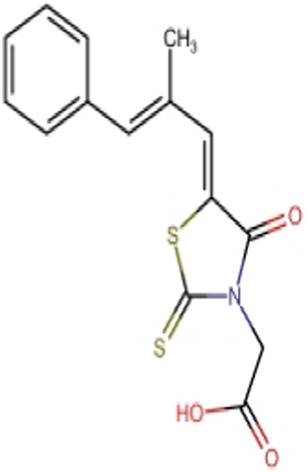 F Epalrestat

## 4 Discussion

The use of natural bioactive compounds including flavones, flavonoids, and coumarins derived from naturally occurring plants, for aldose reductase enzyme inhibition has gained traction in recent years. Numerous studies have investigated the inhibitory effects of synthetic and natural compounds on aldose reductase to mitigate the chronic complications of diabetes, such as nephropathy, retinopathy, and neuropathy (Gamal and Munusamy, 2017; Dănilă et al., 2024). Compounds like epalrestat, sorbinil, tolrestat, and fidarestat have demonstrated significant inhibitory activity against the enzyme in animal models. However, many of these compounds were withdrawn from the market due to adverse effects and lack of selectivity for enzymes sharing sequence homology with aldose reductase, such as aldehyde reductase (Antony and Vijayan, 2015). Among them, only epalrestat, a synthetic aldose reductase inhibitor has successfully undergone clinical trials and is commercially available for treating diabetic neuropathy in Japan and other regions (Zhu and Zhu, 2013). Given the rising prevalence of diabetic complications worldwide, there is an urgent need for alternative and more effective aldose reductase inhibitors to manage diabetic complications. This study is thus aimed to identify natural compounds from the African region with inhibitory activity against aldose reductase.

After screening the pre-filtered library of 2,968 compounds of African origin against aldose reductase (PDB: 1US0), the predicted binding affinities ranged from −12.3 to −3.6 kcal/mol, showcasing a wide range of interaction strengths with the aldose reductase protein. This range highlights the diversity of the compound library and the ability of the docking protocol to discern variations in binding strengths. Among the standard inhibitors screened, zopolrestat, a known aldose reductase inhibitor, had the highest binding affinity of −9.9 kcal/mol and was used as a benchmark. Out of the screened library, 105 compounds displayed binding affinities equal to or better than zopolrestat. This represents approximately 3.5% of the screened compounds, indicating a stringent selection process. To ensure computational feasibility, the top 20 compounds were prioritized for further analysis. These compounds demonstrated binding affinities ranging from −12.3 to −10.7 kcal/mol, significantly outperforming zopolrestat. Such a clear improvement over the benchmark compound indicates the potential of these molecules as aldose reductase inhibitors.

The selected standard inhibitors of aldose reductase include epalrestat, IDD594, sorbinil, tolrestat, and zopolrestat with respective binding affinities of −8.8, −8.1, −7.4, −7.6, and −9.9 kcal/mol, and have been shown to have considerably high inhibitory activity both *in vitro* and *in vivo*. Based on bioactivity studies, sorbinil has been shown to have an IC50 of 3.14 μM (Shehzad et al., 2021). In a study involving streptozotocin diabetic mice, zopolrestat was studied to inhibit at a low IC50 of 0.004 μM (Mylari et al., 2003). IDD594 was studied to have an IC50 of 0.030 μM and is known to be an effective inhibitor of AR (Podjarny et al., 2004). Tolrestat has been studied to have an effective IC50 of 0.0012 µM in a streptozotocin-induced diabetic rat model (Van Zandt et al., 2005). These studies suggest that these standards exhibit notable inhibitory effects on aldose reductase. Nonetheless, the selected top 20 compounds had a higher binding affinity for AR binding sites than all the standard ARIs used in this study highlighting a high potential of the selected compounds as an ARI.

To develop innovative therapeutic agents, it is important to have a thorough understanding of the complex pharmacokinetic dynamics, thereby elucidating the compound’s behavior within the biological environment. This process involves assessing Absorption, Distribution, Metabolism, Excretion, and Toxicity (ADMET) parameters to screen compounds for favorable physicochemical properties (Flores-Holguín et al., 2021; Tian et al., 2015). In this study, compounds exhibiting favorable solubility, pharmacokinetic profiles, and toxicity profiles were meticulously selected. Based on these stringent criteria, eight compounds, namely, (+)-pipoxide, Zinc000095485961, Naamidine A, Sigmoidin-b-4′-methylether diacetate, (−)-pipoxide, (+)-strigol, Isosamarcandin, and 1,6-di-o-p-hydroxybenzoyl-beta-d-glucopyranoside, were identified as possessing drug-like properties with suitable pharmacokinetics and low toxicity. These compounds performed better than some of the standard inhibitors namely, sorbinil, tolrestat, and zopolrestat used in this study.

Intermolecular interactions like hydrogen bonding and hydrophobic interactions play pivotal roles in stabilizing energetically favorable ligands within the open conformational environment of protein structures (Coimbra et al., 2020; Varma et al., 2010). These interactions enhance ligand stability at the target site, influencing binding affinity and drug efficacy (Lou and Martin, 2021). Specifically, (+)-pipoxide, Zinc000095485961, Naamidine A, (−)-pipoxide, and 1,6-di-o-p-hydroxybenzoyl-beta-d-glucopyranoside were chosen from the 8 compounds due to their interaction with specific active site residues of aldose reductase through multiple hydrogen bonds (Coimbra et al., 2020). Hydrogen bonding is essential in drug design, influencing structural stability, enzyme catalysis, and drug partitioning and permeability. Functional groups capable of forming hydrogen bonds in a drug increase their ability to interact with biomolecular targets, enhancing binding and selectivity (Coimbra et al., 2020). Hydrophobic interactions significantly improve inhibitor affinity and selectivity in drug design, with even minor modifications such as adding a methyl group yielding substantial effects (Lou and Martin, 2021). The Hydroxyl groups, benzene rings, and glycosidic linkages of these compounds catalyze the hydrogen bonding and the interactions with the catalytic residues of AR (Lou and Martin, 2021). Zinc000095485961 and Naamidine A’s glycosidic linkage and hydroxyl groups enable strong hydrogen bonding with His110 and Tyr48 (Kingsley et al., 2013) while 1,6-di-o-p-hydroxylbenzoyl-beta-D-glucopyranoside benzene ring and hydroxyl group catalyzes hydrophobic interactions with Trp111 and Trp20. The study showed that the selected compounds have stronger interactions in the AR binding site making them potentially higher inhibitors than the known drug epalrestat.

The integration of PASS and structural similarity analysis provided valuable insights into the biological potential of selected compounds. Our study focused on aldose reductase inhibition, antidiabetic, anti-inflammatory, and antioxidant properties, essential for managing diabetes by addressing glycemic control, oxidative stress, and inflammation. Zinc000095485961 and 1,6-di-o-p-hydroxybenzoyl-beta-D-glucopyranoside were predicted to possess all four activities. Zinc000095485961, identified as eutigoside A, is a natural product from *Stereospermum acuminatissimum*, used for its hemostatic and antiseptic properties in African countries (Leutcha et al., 2023; Sob et al., 2011). Its antidiabetic potential may be attributed to eutigoside A (Kingsley et al., 2013). High structural similarity to drug molecules such as acteoside and echinacoside further supports its potential for drug development. Similarly, 1,6-di-o-p-hydroxybenzoyl-beta-D-glucopyranoside, derived from *Tabebuia* species, has been studied to demonstrate antioxidant, anti-inflammatory, and antidiabetic properties (Govindappa et al., 2013; Murugan et al., 2017; Jimenez-Gonzalez et al., 2018). The lead compound (−)-Pipoxide, from *Uvaria dependens* and *Uvaria dependensis*, traditionally used to treat malaria, exhibited anti-inflammatory, antifungal, antioxidant, and antiviral properties (Nkunya et al., 1993; Mayeka et al., 2024). Similarly, its enantiomer, (+)-pipoxide, derived from *Monanthotaxis buchananii*, displayed similar properties (Liang et al., 1988; Mayeka et al., 2024). While Naamidine A did not exhibit predicted biological activity in our selected parameters, it has been extensively reported for its anticancer activity (LaBarbera et al., 2009; Vaden et al., 2019; LaBarbera et al., 2009; Vaden et al., 2019). Also, (+)-pipoxide, (−)-pipoxide and Naamidine-A showed no significant structural resemblance to known drugs. This highlights the novelty of these compounds and the need for further exploration and testing.

To further validate the selected compounds as lead candidates in this study, we performed molecular dynamics simulations (MDS). MDS serves as a computational tool to elucidate the impact of atomic alterations within a molecular system (Knapp et al., 2011). These simulations provide insights into atomic movements and intermolecular interactions over time, capturing the dynamic behavior and positional changes of atoms (Adelusi et al., 2022). Post-simulation analysis conducted, such as Root Mean Square Deviations (RMSD), Root Mean Square Fluctuations (RMSF), Radius of Gyration (Rg), and hydrogen bonds offer valuable insights into the stability of protein-ligand complexes throughout the simulation period (Arthur et al., 2024; Hanson et al., 2025). These analyses are pivotal for assessing their functional reliability within living systems, thereby influencing the efficacy of drug candidates, a critical aspect in drug discovery (Adelusi et al., 2022; Ashley et al., 2024). In our molecular dynamics (MD) analysis, we compared selected compounds to the standard aldose reductase inhibitor, epalrestat, which is clinically approved for treating diabetic peripheral neuropathy symptoms (Zhu and Zhu, 2013; Ramirez and Borja, 2008).

The Root Mean Square Deviation (RMSD) serves as an indicator of the structural stability of the aldose reductase (AR)-ligand complex (Kuzmanic and Zagrovic, 2010). This study revealed a consistent RMSD profile throughout the 100 ns simulation, suggesting that all the selected compounds maintain the structural stability of the protein upon binding. Literature suggests that RMSD values between 0.15 and 0.25 nm indicate enhanced stability (Arnittali et al., 2021), further emphasizing the stable conformation of all five selected compounds having an average RMSD below 0.2 nm. A stable conformation results in increased affinity to AR thereby increasing the efficacy as a drug (Maruyama et al., 2023). Furthermore, the Radius of Gyration (Rg) analysis highlighted stable protein compactness, indicating that the ligands effectively maintain the protein’s compact conformation upon binding. A fluctuating Rg reveals the lack of compactness which may be due to protein unfolding (Jiang et al., 2019; Kwofie et al., 2022). In this study, all compounds showed a stable Rg and did not differ from the unbound protein which means that the ligands can maintain the compact conformation of aldose reductase when bound to the binding site. Similarly, the Root Mean Square Fluctuation (RMSF) analysis elucidates changes in conformation induced by compound binding, particularly with specific amino acid residues of the protein (Abdullah et al., 2023). A fluctuating or unstable binding site results in a weak binding which increases the energy required for effective binding, making the interaction thermodynamically unfavorable (Du et al., 2016). The highest fluctuation was between residues 210 to 230. Within this region is the loop region (214–230) which exhibits significant flexibility facilitates the binding of diverse substrates and enables conformational adjustments essential for AR’s functional promiscuity. Notably, the observed fluctuations did not significantly differ from those of the unbound protein and the standard inhibitor, indicating minimal influence of the selected compounds on protein conformation. This finding underscores the stability of the AR-ligand complex and the potential for effective inhibition against AR.

The MM-PBSA was calculated to elucidate the binding free energies of five selected compounds. Results indicated high binding free energies, which validate the docking results as the lead compounds’ high binding free energies coincide with the molecular docking’s high binding affinity. Van der Waals energy and the electrostatic energy of the top-selected compounds show strong indicators of stable binding energy to aldose reductase, with an effect stronger than the standard inhibitor. The polar solvation and SASA energy of the compounds are well-balanced, indicating effective hydrophobic packing and solvent exclusion during their inhibitory activities in biological phenomena with aldose reductase. The top five selected ligands exhibit optimal molecular interactions, characterized by strong binding, specificity, and stability, indicating their potential therapeutic effectiveness in effectively inhibiting aldose reductase.

To effectively inhibit aldose reductase, the selected compounds should dock within the binding site and interact with critical residues involved in protein-ligand interactions. Upon evaluating the energy decomposition and binding interactions of the amino acid residues of AR and the ligand complex, it was observed that there were significant binding interactions with critical residues such as His110, Tyr48, Trp111, Trp20, Phe122, Asp43, Cys298, and Trp219, which have also been previously reported in literature as active site residues of AR (Singh et al., 2021b; Ashik et al., 2022). These interactions highlight important inhibitory mechanisms of the selected compounds on aldose reductase. Comparatively, the energy contribution of the critical residues of the selected five compounds is considerably higher than that of the standard inhibitor highlighting the potential of the selected compounds as effective inhibitors.

## 5 Conclusion and next steps

In this study, out of 7,344 African compounds being studied, (+)-pipoxide, Zinc000095485961, 1,6-di-o-p-hydroxybenzoyl-beta-d-glucopyranoside and Naamidine A, (−)-pipoxide were concluded as the top lead compound with molecular binding to aldose reductase, no toxicity and inhibitory activity against aldose reductase. Molecular docking studies predicted a low binding energy of the successful compounds compared to the standard ARIs, suggesting that the predicted compounds have a higher binding affinity to the aldose reductase enzyme. These compounds were shown to possess strong interactions with the binding site residues through hydrogen bonding as well as hydrophobic interactions. Also, through ADMET studies, these compounds have revealed favorable pharmacokinetic properties with no predicted toxicity. Molecular dynamics simulations validated these findings by producing a lower and stable RMSD and Radius of gyration. Equally, relatively similar fluctuations in RMSF indicate stable amino acid residue conformation when the ligands bind to aldose reductase. MM-PBSA analysis confirmed the docking results and highlighted energies contributed by interacting residues when AR is bound to the selected compound. With epalrestat, a potent known inhibitor used as a standard and a benchmark, this study has strongly emphasized the potential of natural African compounds as potential therapeutics in managing diabetic complications. While computational methods such as molecular docking and dynamics simulations provide valuable insights, they are inherently limited by the potential for false positives in predicting compound efficacy. These computational predictions may not accurately reflect the complex biological interactions *in vivo*, necessitating further validation through experimental studies. *In vitro* and *in vivo* studies are necessary steps to prove the effectiveness of these compounds as potential therapeutic AR inhibitors to mitigate complications in diabetic patients.

## Data Availability

The original contributions presented in the study are included in the article/Supplementary Material, further inquiries can be directed to the corresponding author.

## Abbreviations

ADMET, Absorption, Distribution, Metabolism, Excretion, and Toxicity; AR, Aldose Reductase; ARI, Aldose Reductase Inhibitor; AUC, Area Under the Curve; CHARMM, Chemistry at HARvard Molecular Mechanics; DUD-E, Directory of Useful Decoys, Enhanced; PASS, Prediction of Activity Spectra for Substances; RCSB, Research Collaboratory for Structural Bioinformatics; RMSD, Root Mean Square Deviation; RMSF, Root Mean Square Fluctuation; ROC, Receiver Operating Curve; RoG, Radius of Gyration; SPC, Simple Point Charge; UFF, Universal Force Field; MM-PBSA, Molecular Mechanics Poisson-Boltzmann Surface Area.
