# Supplementary material for: Targeting aldose reductase using natural African compounds as promising agents for managing diabetic complications
Source: Front Bioinform. 2025 Feb 6;5:1499255. doi: 10.3389/fbinf.2025.1499255 (PMC11848289; doi:10.3389/fbinf.2025.1499255)
Supplement: Supplementary file 1 [file DataSheet1.docx]

Supplementary Material

# Supplementary Table

Table 1. Top compounds with binding affinity greater than -9.9 (kcal/mol) after docking using Autodock Vina.

| Position | Name | Binding Affinity (kcal/mol) |
| --- | --- | --- |
| 1 | 4,5-di-p-trans-coumaroylquinic_acid | -12.3 |
| 2 | (+)-pipoxide | -11.4 |
| 3 | thymelol | -11.4 |
| 4 | ZINC000095485961 | -11.2 |
| 5 | rutamontine | -11.1 |
| 6 | (-)-tingtanoxide | -11 |
| 7 | tricoccin_S13_acetate | -11 |
| 8 | lactupicrin | -11 |
| 9 | naamidine_A | -11 |
| 10 | ZINC000000134782 | -10.9 |
| 11 | sigmoidin_B_4'-methylether_diacetate | -10.9 |
| 12 | (-)-pipoxide | -10.9 |
| 13 | abyssinone_II | -10.8 |
| 14 | (+)-strigol | -10.8 |
| 15 | norisojamicin | -10.8 |
| 16 | calopogonium_isoflavone_B | -10.8 |
| 17 | isosamarcandin | -10.8 |
| 18 | (+)-pipoxide-2-methyl_ether | -10.8 |
| 19 | ZINC000095485890 | -10.8 |
| 20 | 1,6-di-O-p-hydroxybenzoyl-beta-D-glucopyranoside | -10.7 |
| 21 | ZINC000095486276 | -10.7 |
| 22 | enterolactone | -10.7 |
| 23 | ZINC000095486323 | -10.7 |
| 24 | 6,7-dihydroxykaratavicinol | -10.7 |
| 25 | 8alpha-hydroxy-3beta-(benzoyloxy)-1alphaH,5alphaH,6betaH,7alphaH-guai-4(15),10(14),11(13)-trien-6,12-olide | -10.7 |
| 26 | ferulsinaic_acid | -10.7 |
| 27 | 3-(10-hydroxygeranyl)-4-hydroxy-p-coumaric_acid | -10.7 |
| 28 | monathadiepoxide_methyl_ether | -10.7 |
| 29 | coladin | -10.7 |
| 30 | diversinin | -10.7 |
| 31 | ZINC000013374323 | -10.6 |
| 32 | teucrin_F | -10.6 |
| 33 | durmillone | -10.6 |
| 34 | coladonin | -10.6 |
| 35 | azadironolide | -10.6 |
| 36 | polyanthin | -10.6 |
| 37 | 13-hydroxyfeselol_diacetate | -10.5 |
| 38 | feselol | -10.5 |
| 39 | neveskone | -10.5 |
| 40 | crotohalimaneic_acid | -10.5 |
| 41 | naamidine_G | -10.5 |
| 42 | ZINC000038658035 | -10.5 |
| 43 | megalocarpoidolide_C | -10.4 |
| 44 | isoarnottinin-4'-O-beta-D-glucoside | -10.4 |
| 45 | toussaintine_D | -10.4 |
| 46 | 4beta-hydroxy-6alpha-benzoyl-7-daucen-9-one | -10.3 |
| 47 | desmosdomutin | -10.3 |
| 48 | isojamaicin | -10.3 |
| 49 | 4beta,8beta-dihydroxy-6alpha-p-hydroxybenzoyl-dauc-9-ene | -10.3 |
| 50 | (+)-piperitol-3,3-dimethylallyl_ether | -10.3 |
| 51 | silybin_A | -10.3 |
| 52 | naamidine_D | -10.3 |
| 53 | N-E-caffeoyl_tyramine | -10.3 |
| 54 | farnesiferol_A | -10.3 |
| 55 | 4beta-hydroxy-6alpha(p-hydroxybenzoyloxy)-10alpha-angeloxydauc-7-ene | -10.3 |
| 56 | 3-oxo-gamma-costic_acid_beta-D-glucopyranoside_ester | -10.3 |
| 57 | marmaricin | -10.2 |
| 58 | ZINC000014811038 | -10.2 |
| 59 | ZINC000014557836 | -10.2 |
| 60 | 12'-hydroxygymnastatin_N | -10.2 |
| 61 | diphenylpentanoid_(E)-1,5-bis(4-hydroxyphenyl)-pent-1-en-3-one | -10.2 |
| 62 | (-)-5-hydroxyprantschimgin | -10.1 |
| 63 | ZINC000014618437 | -10.1 |
| 64 | englerin_B | -10.1 |
| 65 | 7,7'-dihydroxy-6,8'-bicoumarin | -10.1 |
| 66 | ZINC000100003416 | -10.1 |
| 67 | daphenone | -10.1 |
| 68 | 3'-prenylnaringenin | -10.1 |
| 69 | diversin | -10.1 |
| 70 | N1,N8-dibenzoylspermidine | -10 |
| 71 | 5-p-trans-coumaroylquinic_acid | -10 |
| 72 | ZINC000100003068 | -10 |
| 73 | (3S)-3,7-dihydroxy-8-methoxy-3-(3',4'-methylenedioxybenzyl)chroman-4-one | -10 |
| 74 | assafoetidnol_A | -10 |
| 75 | p-hydroxyphenethyl_trans-ferulate | -10 |
| 76 | lanceolatin_B | -10 |
| 77 | 3-O-acetyl_chlorogenic_acid | -10 |
| 78 | (-)-5-hydroxydeltoin | -10 |
| 79 | (-)-semiglabrin | -10 |
| 80 | ZINC000032296267 | -10 |
| 81 | enterofuran | -10 |
| 82 | S-trans-marmin | -10 |
| 83 | 8-C-p-hydroxybenzylkaempferol | -10 |
| 84 | antheindurolide_A | -10 |
| 85 | ZINC000006091807 | -10 |
| 86 | paulownia | -10 |
| 87 | 7-[(3'Z,5'E)-7'-hydroxy-3',7'-dimethyl-3',5'-octadienyloxy]coumarin | -10 |
| 88 | sigmoidin_B | -10 |
| 89 | ent-3-hydroxyatis-16(17)-ene-2,14-dione | -10 |
| 90 | transtaganolide_A | -9.9 |
| 91 | citrinamide_A | -9.9 |
| 92 | Zopolrestat | -9.9 |
| 93 | obovatin | -9.9 |
| 94 | 8-C-p-hydroxybenzylapigenin | -9.9 |
| 95 | 4-hydroxy-7-methoxyflavan | -9.9 |
| 96 | antheindurolide_B | -9.9 |
| 97 | uguenenazole | -9.9 |
| 98 | pinobanksin_3-(E)-caffeate | -9.9 |
| 99 | S-(-)-trans-N-feruloyloctopamine | -9.9 |
| 100 | jamaicin | -9.9 |
| 101 | samarcandin | -9.9 |
| 102 | chlorogenoquinone | -9.9 |
| 103 | sarcophytolide | -9.9 |
| 104 | uguenensene | -9.9 |
| 105 | trans-N-feruloyltyramine | -9.9 |

# Supplementary Images

**
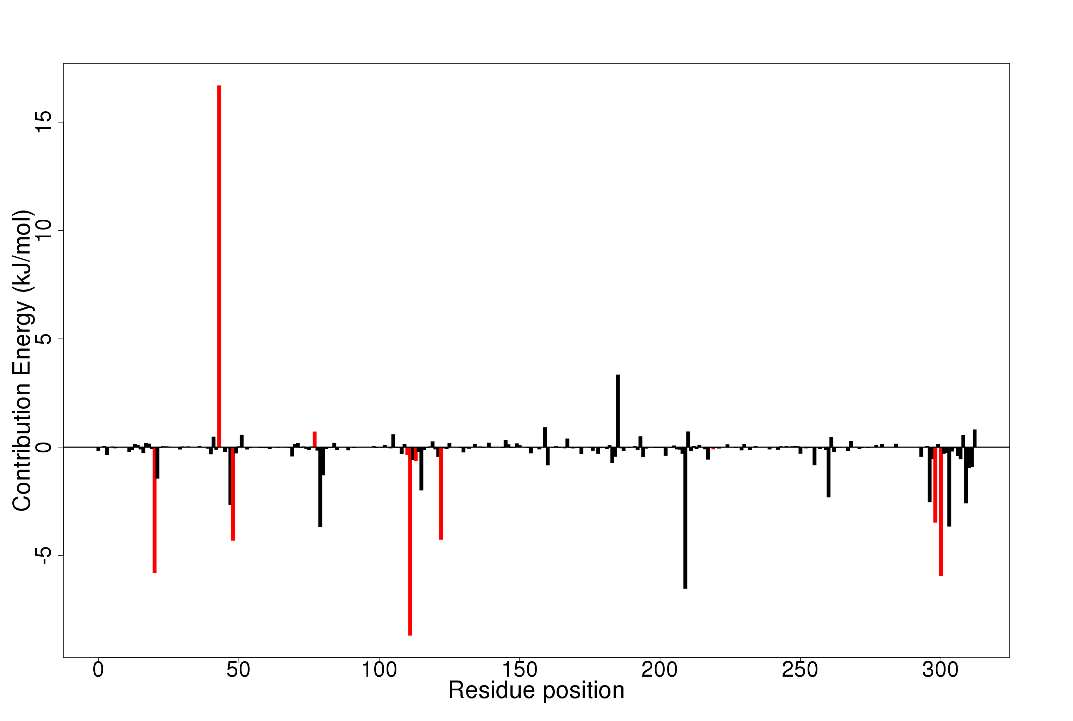
**

**Supplementary Figure 1**. Molecular Mechanics Poisson-Boltzmann Surface Area (MM-PBSA) plot of binding free energy contribution per residue of AR- 1,6-di-o-p-hydroxybenzoyl-beta-d-glucopyranoside complex. Fluctuations by selected critical residues of AR are shown in red.

**
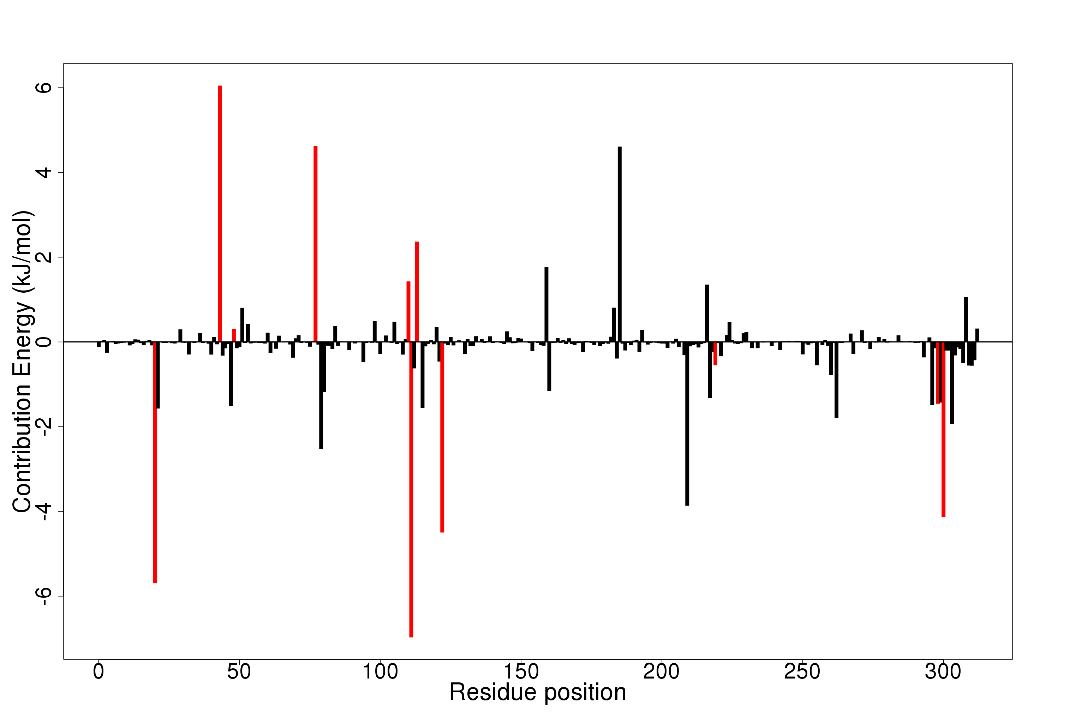
**

**Supplementary Figure 2**. Molecular Mechanics Poisson-Boltzmann Surface Area (MM-PBSA) plot of binding free energy contribution per residue of AR- (+)-pipoxide complex. Fluctuations by selected critical residues of AR are shown in red.

**
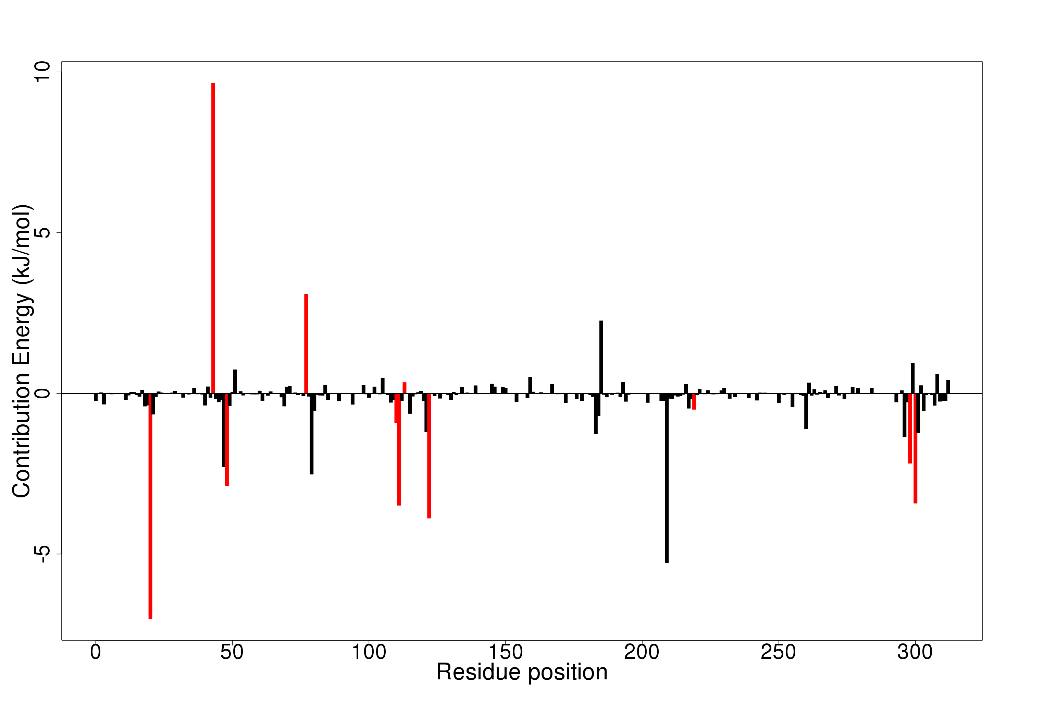
**

**Supplementary Figure 3**. Molecular Mechanics Poisson-Boltzmann Surface Area (MM-PBSA) plot of binding free energy contribution per residue of AR- (-)-pipoxide complex. Fluctuations by selected critical residues of AR are shown in red.

**
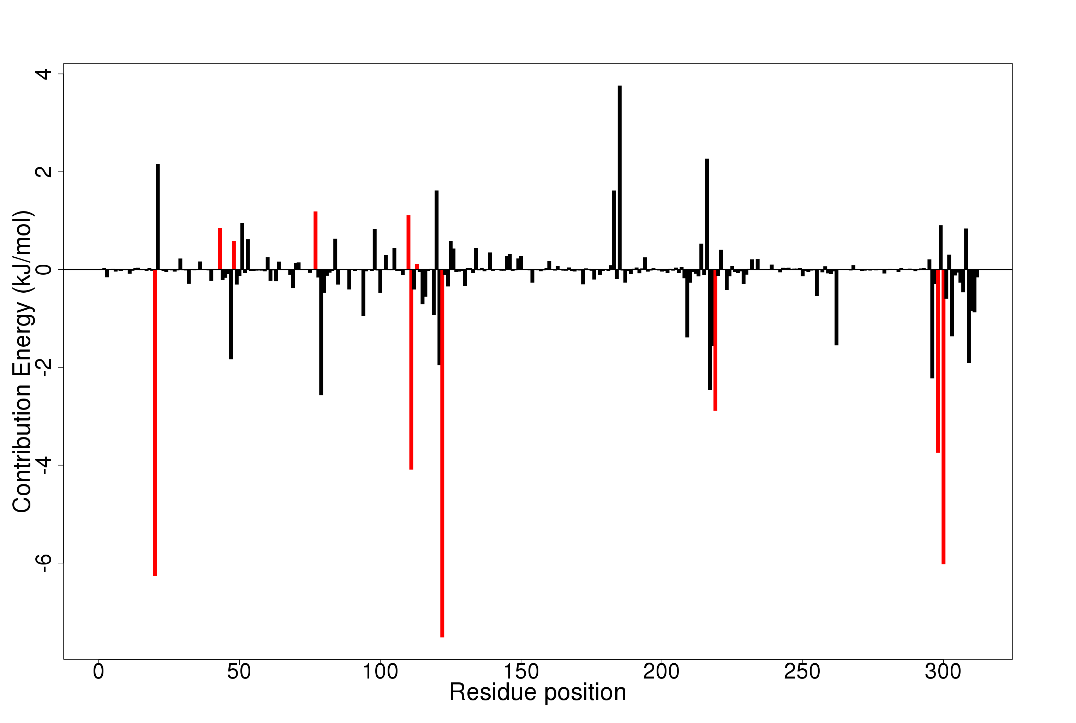
**

**Supplementary Figure 4**. Molecular Mechanics Poisson-Boltzmann Surface Area (MM-PBSA) plot of binding free energy contribution per residue of AR- Naamidine A complex. Fluctuations by selected critical residues of AR are shown in red.


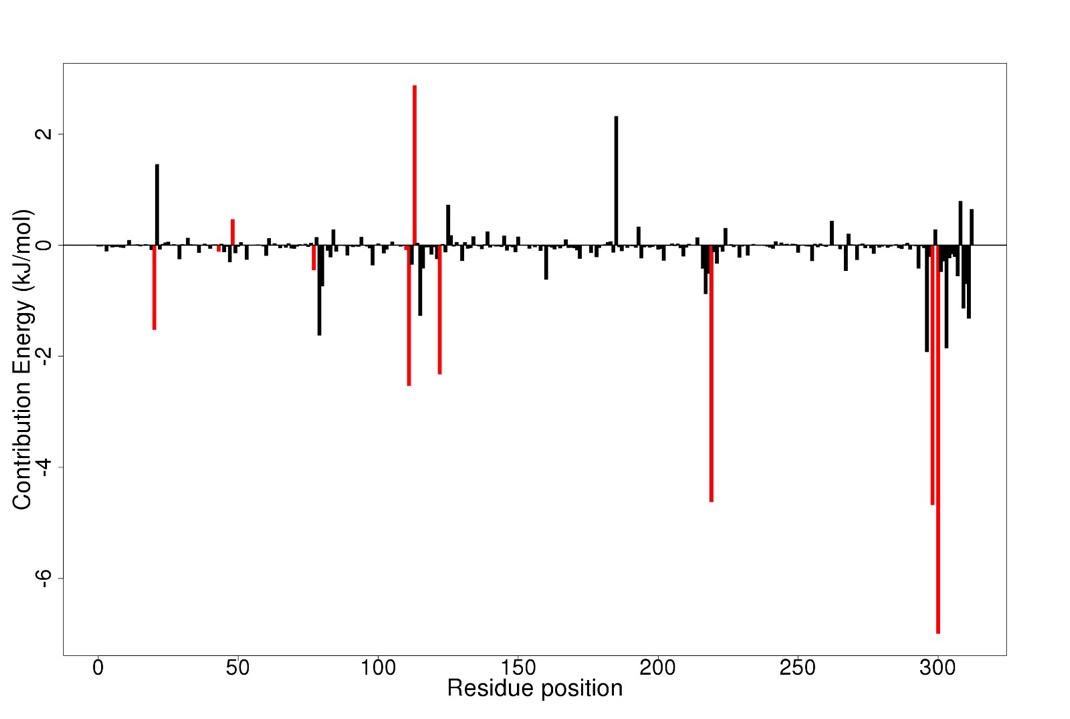


**Supplementary Figure 5**. Molecular Mechanics Poisson-Boltzmann Surface Area (MM-PBSA) plot of binding free energy contribution per residue of AR- Epalrestat complex. Fluctuations by selected critical residues of AR are shown in red.
